# Editorial: Deep learning and neuroimage processing in understanding neurological diseases

**DOI:** 10.3389/fncom.2024.1523973

**Published:** 2024-12-19

**Authors:** Joana Carvalho, Ali Abdollahzadeh, Ricardo José Ferrari

**Affiliations:** ^1^Laboratory of Visual Neuroscience, Faculty of Psychology and Educational Sciences, University of Coimbra, Coimbra, Portugal; ^2^Laboratory of Preclinical MRI, Champalimaud Research, Champalimaud Centre for the Unknown, Lisbon, Portugal; ^3^A. I. Virtanen Institute for Molecular Sciences, University of Eastern Finland, Kuopio, Finland; ^4^Center for Biomedical Imaging, Department of Radiology, New York University School of Medicine, New York, NY, United States; ^5^Biomedical Image Processing (BIP) Lab, Department of Computing, Federal University of São Carlos, São Carlos, Brazil

**Keywords:** deep learning, segmentation, classification, neuroimaging, neurological disorder

## Introduction

Recent advancements in neuroimaging have been instrumental in improving the diagnosis, treatment planning, and monitoring of neurological diseases (Qiu et al., 2020). DL-powered algorithms now excel in tasks like brain segmentation, lesion detection, cohort classification, and biomarker discovery, offering unprecedented insights into high-resolution imaging data. However, challenges like neuroimaging data variability and quality, limited training on pathological cases, and poor generalizability across modalities persist (Ma et al., 2024). This Research Topic addresses these challenges, presenting recent DL advancements in neuroimaging analysis, including enhancements in brain segmentation accuracy, refined cohort classification strategies, improved anatomical feature extraction, and investigations of gender differences in Neuroimaging data (CT, MRI, and EEG). The goal is to develop more robust and generalizable models that can accommodate the inherent variability in neuroimaging datasets, ultimately improving clinical decision-making and patient outcomes.

## Deep learning methods to improve biomedical image segmentation

The segmentation of neuroimaging data plays a crucial role in treatment and surgical planning, diagnosis, and monitoring (Isensee et al., 2021). Image segmentation involves identifying and outlining structural and functional regions of interest (ROIs), lesions, vessels, or white matter tracts, among others. Although manual segmentation remains the ground truth, it is highly time-consuming, labor-intensive, requires expert knowledge, and is prone to variability between observers and even within the same observer over time (Haque and Neubert, 2020). To address these limitations, semi-automatic and fully automatic segmentation methods offer significant advantages, reducing time and labor requirements, enhancing consistency, and enabling the efficient analysis of large-scale datasets (Antonelli et al., 2022). However, most DL models for segmentation require labeled training data obtained through experts' manual annotation.

To address this, Duarte et al. introduced a multi-stage semi-supervised learning (M3SL) approach to segment white matter hyperintensities in MRI T1-weighted anatomical scans automatically. The M3SL method employs a three-step optimization process, leveraging unannotated data segmented by traditional processing methods. This approach has been shown to outperform both conventional baseline methods and deep learning models based on transfer learning. The M3SL method demonstrates resilience against annotation scarcity, offering a robust alternative for semi-supervised segmentation.

The generalizability of DL models for automatic segmentation can be improved by retraining them on data from pathological cases. For instance, Gerken et al., demonstrated that including hemorrhage data in the training set improved the automatic segmentation of brain parenchyma and cerebrospinal fluid, i.e., ventricles, in CT images. In this study, Gerken et al. first trained a 2D U-Net (Ronneberger et al., 2015) on brain scans of healthy participants and subsequently retrained it with additional hemorrhage data. The model was tested on datasets that included normal, hemorrhage, and tumor cases. Adding hemorrhage data significantly improved segmentation performance, not only on hemorrhage cases but also on tumor cases, without negatively impacting performance on normal data. This study shows that expanding the training set to include a broader range of pathologies may improve generalizability.

## Deep learning methods to categorize cohorts based on neuroimaging

The capacity of DL to automatically extract and learn complex patterns from vast and diverse datasets has made it a transformative tool for cohort classification in biomedical research. DL models can accurately analyze and classify images, detecting subtle features that distinguish between healthy individuals and those with specific conditions (Chan et al., 2020). This capability enables critical tasks in disease diagnosis, prognosis, and predicting treatment response across a range of medical imaging modalities.

One prominent application of DL is analyzing EEG data to detect epileptic seizures (Gao et al., 2020). EEG is the preferred neuroimaging modality for seizure detection; however, its strong spatial and temporal correlations pose challenges for accurate classification. To address this, Hu et al. introduced an innovative epileptic seizure classification model based on Iterative Gated Graph Convolutional Networks (IGGCN). This model effectively captures long-term dependencies in EEG data, resulting in significant classification performance improvements. By addressing the complexities of EEG signal analysis, the IGGCN advances the accuracy and reliability of seizure detection, enhancing DL's role in neuroimaging-based diagnostics.

Similarly, DL techniques also encounter unique challenges in pediatric neuroimaging. Pediatric brain development is rapid and dynamic, and neuroimaging data is often of lower quality due to motion artifacts, compounded by the limited availability of age-specific, high-quality datasets. To address these obstacles, Das et al. used a DL model to detect prenatal alcohol exposure in children. They applied a pre-trained simple fully convolutional network for feature extraction and trained a classifier to differentiate between exposed and unexposed children using MRI scans. This approach minimized reliance on large datasets, boosting generalizability through data augmentation. The classifier achieved high sensitivity and accuracy, with key brain regions, such as the corpus callosum, cerebellum, pons, and white matter, identified as predictive for clinical decision-making. This underscores the valuable role of DL in pediatric imaging.

DL techniques have also been applied to investigate sex differences in brain MRI data. For example, Dibaji et al. used convolutional neural networks to classify gender with high accuracy and minimal image preprocessing. Saliency maps were employed to identify key brain regions contributing to sex differentiation, offering valuable insights into sex-specific anatomical differences. This research not only improves our understanding of brain structure but also suggests strategies to mitigate bias in AI models, ultimately contributing to fairer healthcare outcomes.

## Conclusion

This Research Topic highlights innovative DL approaches enhancing tissue segmentation, biomarker identification, and early diagnosis of neurological conditions like epilepsy, cognitive impairment, brain lesions, and prenatal alcohol exposure. The models improve generalizability, reduce reliance on annotated data, and deliver more accurate segmentation and pathology detection. These findings promise to accelerate the development of diagnostic tools and enable more personalized treatment plans for neurologic conditions, offering significant potential to improve patient outcomes.
